# Supplementary material for: Prestimulus functional connectivity reflects attention orientation in a prospective memory task: A magnetoencephalographic (MEG) study
Source: PLoS One. 2025 Feb 25;20(2):e0319213. doi: 10.1371/journal.pone.0319213 (PMC11856308; doi:10.1371/journal.pone.0319213)
Supplement: S1 Table — Table displaying correspondences between each Region of Interest (Destrieux Atlas) and the corresponding Yeo Network. Coordinates indicate the centroid of each region. (DOCX) [file pone.0319213.s001.docx]

**S1 Table. Correspondence between Destrieux regions and Yeo Networks.**

Table displaying correspondences between each Region of Interest (Destrieux Atlas) and the corresponding Yeo Netwok. Coordinates indicate the centroid of each region.

| Region of interest (Name) | Destrieux Labels | Yeo Network | Coordinates | | |
| --- | --- | --- | --- | --- | --- |
|  |  |  | X | Y | Z |
|  |  |  |  |  |  |
| Anterior Cingulate cortex (left) | l.G_and_S_cingul-Ant | Default Mode | -10877 | 39073 | 8334 |
| Dorsal-Posterior Cingulate cortex (left) | l.G_cingul-Post-dorsal | Default Mode | -5101 | -36409 | 30514 |
| Ventral-Posterior Cingulate cortex (left) | l.G_cingul-Post-ventral | Default Mode | -9817 | -46978 | 5625 |
| Inferior Frontal gyrus - pars opercularis (left) | l.G_front_inf-Opercular | Default Mode | -50085 | 1187 | 746 |
| Inferior Frontal gyrus - pars orbitalis (left) | l.G_front_inf-Orbital | Default Mode | -44306 | 30741 | -10499 |
| Inferior Frontal gyrus - pars triangularis (left) | l.G_front_inf-Triangul | Default Mode | -48826 | 29933 | 3848 |
| Angular gyrus (left) | l.G_pariet_inf-Angular | Default Mode | -42927 | -64441 | 38345 |
| Subcallosal gyrus (left) | l.G_subcallosal | Default Mode | -5987 | 15553 | -8696 |
| Lateral Superior Temporal gyrus (left) | l.G_temp_sup-Lateral | Default Mode | -59331 | -15346 | -934 |
| Anterior Lateral Fissure, Horizontal ramus (left) | l.Lat_Fis-ant-Horizont | Default Mode | -3882 | 32499 | -2872 |
| Anterior Lateral Fissure, Vertical ramus (left) | l.Lat_Fis-ant-Vertical | Default Mode | -43715 | 21733 | 8441 |
| Anterior Circular sulcus (left) | l.S_circular_insula_ant | Default Mode | -27738 | 22278 | -9516 |
| Superior Frontal sulcus (left) | l.S_front_sup | Default Mode | -22711 | 17928 | 43159 |
| Sulcus Intermedius Primus (left) | l.S_interm_prim-Jensen | Default Mode | -49911 | -5226 | 30213 |
| Lateral Orbital sulcus (left) | l.S_orbital_lateral | Default Mode | -3946 | 40593 | -229 |
| Suborbital sulcus (left) | l.S_suborbital | Default Mode | -8559 | 38452 | -11471 |
| Subparietal sulcus (left) | l.S_subparietal | Default Mode | -10867 | -5005 | 34979 |
| Anterior Cingulate cortex (right) | r.G_and_S_cingul-Ant | Default Mode | 11187 | 38601 | 8006 |
| Ventral-Posterior Cingulate cortex (right) | r.G_cingul-Post-ventral | Default Mode | 10218 | -49055 | 738 |
| Inferior Frontal gyrus - pars orbitalis (right) | r.G_front_inf-Orbital | Default Mode | 47925 | 3235 | -6988 |
| Inferior Frontal gyrus - pars triangularis (right) | r.G_front_inf-Triangul | Default Mode | 5105 | 28051 | 6786 |
| Parahippocampal gyrus (right) | r.G_oc-temp_med-Parahip | Default Mode | 25122 | -16652 | -2774 |
| Anterior Lateral Fissure, Vertical ramus (right) | r.Lat_Fis-ant-Vertical | Default Mode | 45448 | 21327 | 9036 |
| Anterior Circular sulcus (right) | r.S_circular_insula_ant | Default Mode | 29668 | 23312 | -7027 |
| Anterior Transverse Collateral sulcus (right) | r.S_collat_transv_ant | Default Mode | 41753 | -20346 | -24345 |
| Superior Frontal sulcus (right) | r.S_front_sup | Default Mode | 2269 | 18148 | 44827 |
| Sulcus Intermedius Primus (right) | r.S_interm_prim-Jensen | Default Mode | 47946 | -44615 | 35704 |
| H-shaped Orbital sulci (right) | r.S_orbital-H_Shaped | Default Mode | 24293 | 35417 | -11756 |
| Suborbital sulcus (right) | r.S_suborbital | Default Mode | 8781 | 42174 | -14271 |
| Subparietal sulcus (right) | r.S_subparietal | Default Mode | 10497 | -50187 | 36502 |
| Fusiform gyrus (left) | l.G_oc-temp_lat-fusifor | Dorsal Attention | -35646 | -53889 | -17966 |
| Superior parietal lobule (left) | l.G_parietal_sup | Dorsal Attention | -18662 | -5773 | 58569 |
| Lateral occipito-temporal sulcus (left) | l.S_oc-temp_lat | Dorsal Attention | -4184 | -49887 | -14185 |
| Anterior Occipital sulcus (left) | l.S_occipital_ant | Dorsal Attention | -417 | -68154 | 278 |
| Postcentral sulcus (left) | l.S_postcentral | Dorsal Attention | -36815 | -36102 | 41146 |
| Superior Precentral sulcus (left) | l.S_precentral-sup-part | Dorsal Attention | -26797 | -9499 | 50401 |
| Inferior Occipital gyrus and sulcus (right) | r.G_and_S_occipital_inf | Dorsal Attention | 42104 | -76432 | -11482 |
| Superior parietal lobule (right) | r.G_parietal_sup | Dorsal Attention | 18166 | -53808 | 6008 |
| Lateral occipito-temporal sulcus (right) | r.S_oc-temp_lat | Dorsal Attention | 43127 | -50709 | -13523 |
| Anterior Occipital sulcus (right) | r.S_occipital_ant | Dorsal Attention | 42996 | -65226 | 4085 |
| Postcentral sulcus (right) | r.S_postcentral | Dorsal Attention | 36512 | -34916 | 42321 |
| Superior Precentral sulcus (right) | r.S_precentral-sup-part | Dorsal Attention | 26621 | -868 | 5156 |
| Inferior temporal sulcus (right) | r.S_temporal_inf | Dorsal Attention | 53027 | -3985 | -15285 |
| Fronto-Marginal gyrus and sulcus (left) | l.G_and_S_frontomargin | FrontoParietal Control | -24026 | 53836 | -6229 |
| Transverse Frontopolar gyrus and sulcus (left) | l.G_and_S_transv_frontopol | FrontoParietal Control | -15187 | 61795 | 472 |
| Precuneus (left) | l.G_precuneus | FrontoParietal Control | -7562 | -58501 | 4507 |
| Inferior Frontal sulcus (left) | l.S_front_inf | FrontoParietal Control | -38112 | 22769 | 21882 |
| Intraparietal sulcus (left) | l.S_intrapariet_and_P_trans | FrontoParietal Control | -28528 | -58348 | 38653 |
| Parieto-occipital sulcus (left) | l.S_parieto_occipital | FrontoParietal Control | -16599 | -6745 | 22655 |
| Pericallosal sulcus (left) | l.S_pericallosal | FrontoParietal Control | -6016 | -10328 | 25728 |
| Inferior Precentral sulcus (left) | l.S_precentral-inf-part | FrontoParietal Control | -43352 | 2957 | 27034 |
| Fronto-Marginal gyrus and sulcus (right) | r.G_and_S_frontomargin | FrontoParietal Control | 22974 | 55986 | -8061 |
| Transverse Frontopolar gyrus and sulcus (right) | r.G_and_S_transv_frontopol | FrontoParietal Control | 16678 | 61254 | 1985 |
| Dorsal-Posterior Cingulate cortex (right) | r.G_cingul-Post-dorsal | FrontoParietal Control | 5363 | -36443 | 30714 |
| Inferior Frontal gyrus - pars opercularis (right) | r.G_front_inf-Opercular | FrontoParietal Control | 50918 | 9801 | 8232 |
| Angular gyrus (right) | r.G_pariet_inf-Angular | FrontoParietal Control | 46059 | -60622 | 39301 |
| Precuneus (right) | r.G_precuneus | FrontoParietal Control | 7723 | -59505 | 46822 |
| Middle Temporal gyrus (right) | r.G_temporal_middle | FrontoParietal Control | 59788 | -33921 | -12556 |
| Inferior Frontal sulcus (right) | r.S_front_inf | FrontoParietal Control | 387 | 23248 | 20601 |
| Intraparietal sulcus (right) | r.S_intrapariet_and_P_trans | FrontoParietal Control | 30463 | -56032 | 42846 |
| Lateral Orbital sulcus (right) | r.S_orbital_lateral | FrontoParietal Control | 41491 | 39606 | -1682 |
| Pericallosal sulcus (right) | r.S_pericallosal | FrontoParietal Control | 7329 | -8271 | 2571 |
| Inferior Precentral sulcus (right) | r.S_precentral-inf-part | FrontoParietal Control | 42614 | 4307 | 27246 |
| Parahippocampal gyrus (left) | l.G_oc-temp_med-Parahip | Limbic | -2367 | -1945 | -26838 |
| Orbital gyrus (left) | l.G_orbital | Limbic | -2736 | 30677 | -17425 |
| Gyrus Rectus (left) | l.G_rectus | Limbic | -5893 | 36009 | -20016 |
| Planum Polare of the Superior temporal gyrus (left) | l.G_temp_sup-Plan_polar | Limbic | -37993 | 2343 | -25039 |
| Inferior Temporal gyrus (left) | l.G_temporal_inf | Limbic | -51331 | -37894 | -2464 |
| Middle Temporal gyrus (left) | l.G_temporal_middle | Limbic | -58882 | -35896 | -12614 |
| Temporal Pole (left) | l.Pole_temporal | Limbic | -3489 | 2935 | -37098 |
| Anterior Transverse Collateral sulcus (left) | l.S_collat_transv_ant | Limbic | -40799 | -21096 | -23879 |
| Medial Orbital sulcus (left) | l.S_orbital_med-olfact | Limbic | -12768 | 2456 | -17598 |
| H-shaped Orbital sulci (left) | l.S_orbital-H_Shaped | Limbic | -24206 | 34927 | -11657 |
| Inferior temporal sulcus (left) | l.S_temporal_inf | Limbic | -52545 | -38438 | -16527 |
| Orbital gyrus (right) | r.G_orbital | Limbic | 2658 | 3004 | -1702 |
| Gyrus Rectus (right) | r.G_rectus | Limbic | 654 | 33631 | -20422 |
| Subcallosal gyrus (right) | r.G_subcallosal | Limbic | 6973 | 1534 | -11926 |
| Planum Polare of the Superior temporal gyrus (right) | r.G_temp_sup-Plan_polar | Limbic | 40349 | 3752 | -24303 |
| Inferior Temporal gyrus (right) | r.G_temporal_inf | Limbic | 52585 | -34688 | -23578 |
| Temporal Pole (right) | r.Pole_temporal | Limbic | 38295 | 5021 | -36584 |
| Medial Orbital sulcus (right) | r.S_orbital_med-olfact | Limbic | 13776 | 22903 | -16664 |
| Paracentral lobule and sulcus (left) | l.G_and_S_paracentral | Somatomotor | -7879 | -36062 | 68083 |
| Subcentral sulcus and gyrus (left) | l.G_and_S_subcentral | Somatomotor | -56559 | -11103 | 15503 |
| Postcentral gyrus (left) | l.G_postcentral | Somatomotor | -45544 | -25929 | 54949 |
| Precentral gyrus (left) | l.G_precentral | Somatomotor | -41773 | -972 | 51554 |
| Anterior Transverse Superior Temporal gyrus (left) | l.G_temp_sup-G_T_transv | Somatomotor | -47781 | -21011 | 517 |
| Superior Temporal gyrus, Planum Temporale (left) | l.G_temp_sup-Plan_tempo | Somatomotor | -57002 | -41383 | 15188 |
| Anterior Lateral Fissure, Posterior ramus (left) | l.Lat_Fis-post | Somatomotor | -39745 | -3467 | 18317 |
| Central sulcus (left) | l.S_central | Somatomotor | -36398 | -21981 | 46486 |
| Superior Temporal sulcus (left) | l.S_temporal_sup | Somatomotor | -48624 | -47475 | 5828 |
| Transverse Temporal sulcus (left) | l.S_temporal_transverse | Somatomotor | -50606 | -25294 | 2119 |
| Middle-Posterior Cingulate cortex (right) | r.G_and_S_cingul-Mid-Post | Somatomotor | 9828 | -9724 | 40879 |
| Paracentral lobule and sulcus (right) | r.G_and_S_paracentral | Somatomotor | 6915 | -34854 | 68429 |
| Subcentral sulcus and gyrus (right) | r.G_and_S_subcentral | Somatomotor | 56534 | -10438 | 16768 |
| Postcentral gyrus (right) | r.G_postcentral | Somatomotor | 45826 | -23617 | 54373 |
| Precentral gyrus (right) | r.G_precentral | Somatomotor | 40651 | -8156 | 53418 |
| Anterior Transverse Superior Temporal gyrus (right) | r.G_temp_sup-G_T_transv | Somatomotor | 48106 | -19114 | 5111 |
| Lateral Superior Temporal gyrus (right) | r.G_temp_sup-Lateral | Somatomotor | 60502 | -12851 | -826 |
| Superior Temporal gyrus, Planum Temporale (right) | r.G_temp_sup-Plan_tempo | Somatomotor | 58087 | -34708 | 15135 |
| Anterior Lateral Fissure, Posterior ramus (right) | r.Lat_Fis-post | Somatomotor | 38608 | -28952 | 19349 |
| Central sulcus (right) | r.S_central | Somatomotor | 3589 | -1996 | 4641 |
| Superior Temporal sulcus (right) | r.S_temporal_sup | Somatomotor | 48138 | -43634 | 8103 |
| Transverse Temporal sulcus (right) | r.S_temporal_transverse | Somatomotor | 51842 | -22388 | 4422 |
| Middle-Anterior Cingulate cortex (left) | l.G_and_S_cingul-Mid-Ant | Ventral Attention | -10191 | 13856 | 35766 |
| Middle-Posterior Cingulate cortex (left) | l.G_and_S_cingul-Mid-Post | Ventral Attention | -10189 | -11567 | 39832 |
| Middle Frontal gyrus (left) | l.G_front_middle | Ventral Attention | -3727 | 297 | 31553 |
| Superior Frontal gyrus (left) | l.G_front_sup | Ventral Attention | -903 | 2165 | 51327 |
| Long Insular gyrus and Central Insular sulcus (left) | l.G_Ins_lg_and_S_cent_ins | Ventral Attention | -3646 | -7484 | -58 |
| Short Insular gyrus (left) | l.G_insular_short | Ventral Attention | -35043 | 8586 | -2554 |
| Supramarginal gyrus (left) | l.G_pariet_inf-Supramar | Ventral Attention | -55777 | -36111 | 31904 |
| Marginal branch of the Cingulate sulcus (left) | l.S_cingul-Marginalis | Ventral Attention | -1526 | -37588 | 45383 |
| Inferior Circular sulcus (left) | l.S_circular_insula_inf | Ventral Attention | -39502 | -13872 | -7846 |
| Superior Circular sulcus (left) | l.S_circular_insula_sup | Ventral Attention | -34484 | 5133 | 11951 |
| Middle Frontal sulcus (left) | l.S_front_middle | Ventral Attention | -25968 | 43597 | 18904 |
| Middle-Anterior Cingulate cortex (right) | r.G_and_S_cingul-Mid-Ant | Ventral Attention | 1039 | 14795 | 36112 |
| Middle Frontal gyrus (right) | r.G_front_middle | Ventral Attention | 38204 | 29401 | 30849 |
| Superior Frontal gyrus (right) | r.G_front_sup | Ventral Attention | 928 | 22781 | 52457 |
| Long Insular gyrus and Central Insular sulcus (right) | r.G_Ins_lg_and_S_cent_ins | Ventral Attention | 37305 | -4064 | -3656 |
| Short Insular gyrus (right) | r.G_insular_short | Ventral Attention | 36318 | 9454 | -2202 |
| Supramarginal gyrus (right) | r.G_pariet_inf-Supramar | Ventral Attention | 5653 | -31724 | 30782 |
| Anterior Lateral Fissure, Horizontal ramus (right) | r.Lat_Fis-ant-Horizont | Ventral Attention | 41415 | 30224 | 2684 |
| Marginal branch of the Cingulate sulcus (right) | r.S_cingul-Marginalis | Ventral Attention | 14303 | -3759 | 45995 |
| Inferior Circular sulcus (right) | r.S_circular_insula_inf | Ventral Attention | 41392 | -1295 | -787 |
| Superior Circular sulcus (right) | r.S_circular_insula_sup | Ventral Attention | 34968 | 5708 | 11641 |
| Middle Frontal sulcus (right) | r.S_front_middle | Ventral Attention | 28296 | 4093 | 22288 |
| Inferior Occipital gyrus and sulcus (left) | l.G_and_S_occipital_inf | Visual | -3927 | -81242 | -11977 |
| Cuneus (left) | l.G_cuneus | Visual | -5703 | -82559 | 1505 |
| Lingual gyrus (left) | l.G_oc-temp_med-Lingual | Visual | -11197 | -73654 | -5086 |
| Middle Occipital gyrus (left) | l.G_occipital_middle | Visual | -39239 | -82374 | 11232 |
| Superior Occipital gyrus (left) | l.G_occipital_sup | Visual | -14847 | -86955 | 26707 |
| Occipital Pole (left) | l.Pole_occipital | Visual | -17721 | -97276 | -5706 |
| Calcarine sulcus (left) | l.S_calcarine | Visual | -17114 | -66698 | 4784 |
| Posterior Transverse Collateral sulcus (left) | l.S_collat_transv_post | Visual | -2454 | -78901 | -7608 |
| Middle Occipital and Lunatus sulcus (left) | l.S_oc_middle_and_Lunatus | Visual | -30314 | -85287 | 381 |
| Superior Occipital and Transverse sulcus (left) | l.S_oc_sup_and_transversal | Visual | -26886 | -80465 | 17196 |
| Medial Collateral and Lingual sulcus (left) | l.S_oc-temp_med_and_Lingual | Visual | -30414 | -4815 | -937 |
| Cuneus (right) | r.G_cuneus | Visual | 6428 | -81143 | 17312 |
| Fusiform gyrus (right) | r.G_oc-temp_lat-fusifor | Visual | 3463 | -52534 | -17037 |
| Lingual gyrus (right) | r.G_oc-temp_med-Lingual | Visual | 12425 | -67656 | -446 |
| Middle Occipital gyrus (right) | r.G_occipital_middle | Visual | 40033 | -79453 | 12821 |
| Superior Occipital gyrus (right) | r.G_occipital_sup | Visual | 18948 | -85935 | 31252 |
| Occipital Pole (right) | r.Pole_occipital | Visual | 17394 | -94985 | -3658 |
| Calcarine sulcus (right) | r.S_calcarine | Visual | 1888 | -62432 | 5357 |
| Posterior Transverse Collateral sulcus (right) | r.S_collat_transv_post | Visual | 25687 | -78075 | -8216 |
| Middle Occipital and Lunatus sulcus (right) | r.S_oc_middle_and_Lunatus | Visual | 32802 | -82079 | 6453 |
| Superior Occipital and Transverse sulcus (right) | r.S_oc_sup_and_transversal | Visual | 27821 | -77321 | 20078 |
| Medial Collateral and Lingual sulcus (right) | r.S_oc-temp_med_and_Lingual | Visual | 31511 | -46227 | -10155 |
| Parieto-occipital sulcus (right) | r.S_parieto_occipital | Visual | 17959 | -65668 | 24804 |
