# Supplementary figures and images for: Prestimulus functional connectivity reflects attention orientation in a prospective memory task: A magnetoencephalographic (MEG) study
Source: PLoS One. 2025 Feb 25;20(2):e0319213. doi: 10.1371/journal.pone.0319213 (PMC11856308; doi:10.1371/journal.pone.0319213)

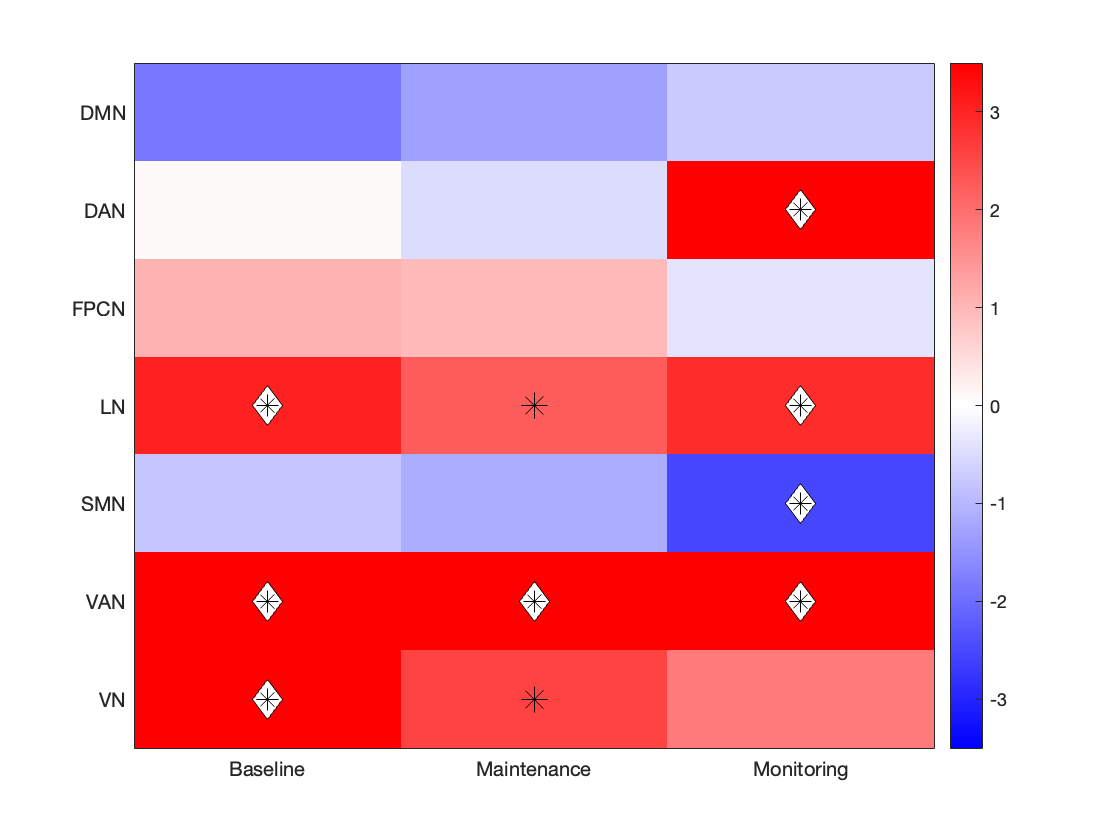

Supplement: S1 Fig — Networks presenting significant differences between InterNC and IntraNC in the alpha band. Differences significant for p < .05 are displayed as black asterisks, whereas those significant for q < .05 are marked with a white diamond. The scale on the left represents t-values derived from statistical comparisons. DMN: Default Mode Network; DAN: Dorsal Attention Network; FPCN: FrontoParietal Control Network; LN: Limbic Network; SMN: SomatoMotor Network; VAN: Ventral Attention Network; VN: Visual Network. (TIF) [file pone.0319213.s002.tif]

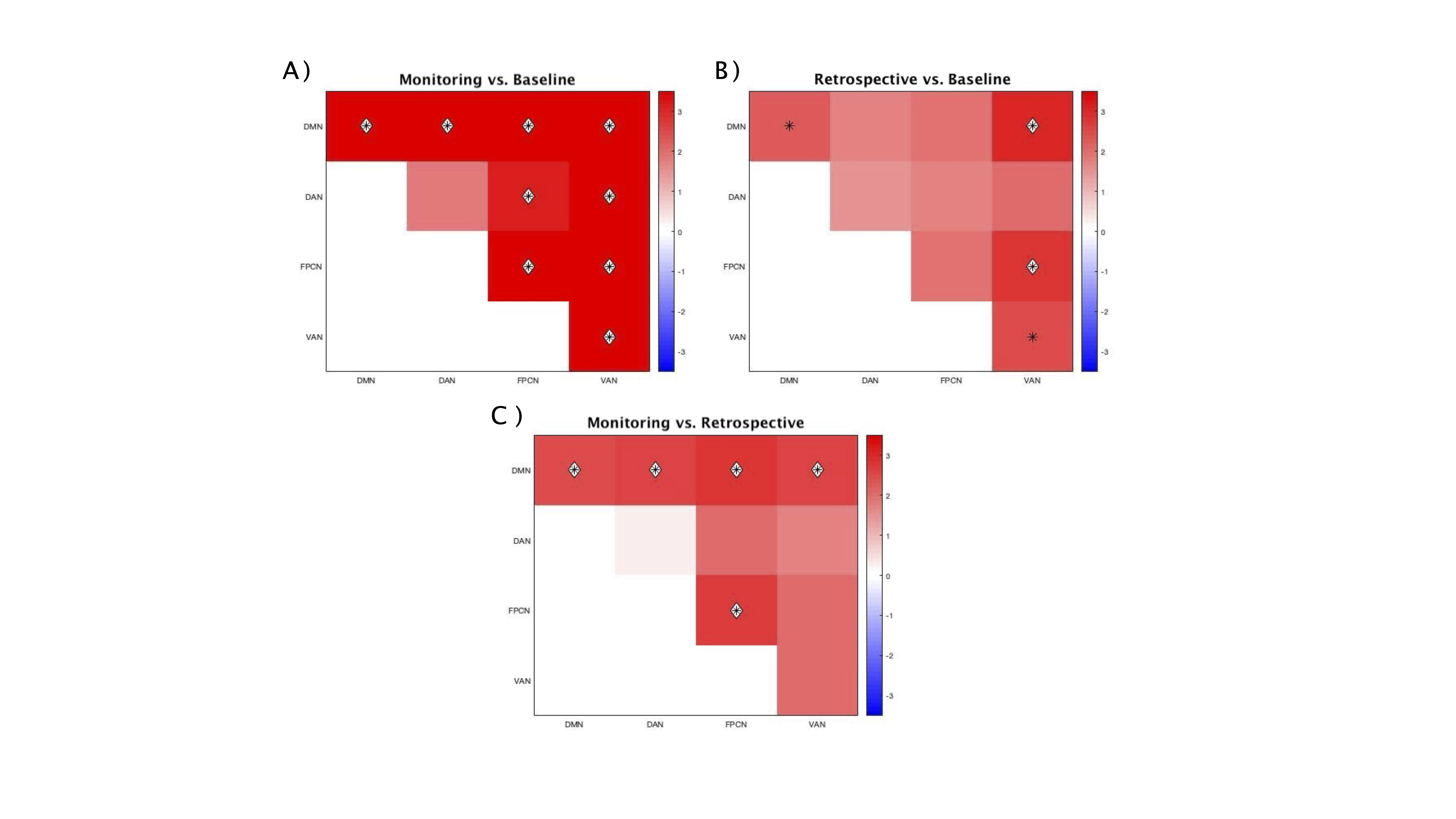

Supplement: S2 Fig — Comparison of Functional Connectivity in the three conditions in the alpha band. A. Comparison between the Monitoring-load and the Baseline conditions; B. Comparison between the Maintenance-load and the Baseline conditions; C. Comparison between the two PM conditions. Differences significant for p < .05 are displayed as black asterisks, whereas those significant for q < .05 are marked with a white diamond. The scale on the left represents t-values derived from statistical comparisons. DMN: Default Mode Network; DAN: Dorsal Attention Network; FPCN: FrontoParietal Control Network; LN: Limbic Network; SMN: SomatoMotor Network; VAN: Ventral Attention Network; VN: Visual Network. (TIF) [file pone.0319213.s003.tif]
