# Supplementary material for: Prestimulus functional connectivity reflects attention orientation in a prospective memory task: A magnetoencephalographic (MEG) study
Source: PLoS One. 2025 Feb 25;20(2):e0319213. doi: 10.1371/journal.pone.0319213 (PMC11856308; doi:10.1371/journal.pone.0319213)
Supplement: Attachment — Submitted filename: Response to Reviewers.docx [file pone.0319213.s005.docx]

To the kind attention of Prof. Farzin Hajebrahimi,

Academic Editor of PLOS ONE

Dear Editor,

We thank you and the Reviewers for the comments and valuable suggestions included in your revisions for the manuscript “Prestimulus functional connectivity reflects attention orientation in a prospective memory task: a magnetoencephalographic (MEG) study" (Manuscript Number: **PONE-D-24-30533**).

We thank the Reviewers for their positive feedback on the interest of our research question and on the quality of our writing. Each one of them raised important and useful concerns on the methods and analyses we applied in this study, and, thanks to their suggestions, we have tried to solve ambiguities and critical aspects in the best way possible. The missing information identified by the Reviewers are now included in the manuscript, and the remaining grammatical errors corrected. Point-by-point responses to Reviewers are reported below in blue font. Changes made to the manuscript are reported below in italic green font and are presented in the tracked version of the manuscript in red font.

All things considered, we hope these modifications and improvements will help the manuscript meet both the Reviewer’s expectations and the publishing standard of PLOS ONE. Thanks again for your consideration of our manuscript for publication in your journal. If you have any questions about this paper, please don’t hesitate to let us know.

We look forward to hearing from you.

With kind regards,

Stefano Vicentin

(on behalf of all authors)

[stefano.vicentin@unipd.it](mailto:vicentinstefano@gmail.com)

**Academic Editor:** Please ensure that your manuscript meets PLOS ONE's style requirements, including those for file naming. The PLOS ONE style templates can be found at

<https://journals.plos.org/plosone/s/file?id=wjVg/PLOSOne_formatting_sample_main_body.pdf> and

[https://journals.plos.org/plosone/s/file?id=ba62/PLOSOne_formatting_sample_title_authors_affiliations.pdf](https://track.editorialmanager.com/CL0/https:%2F%2Fjournals.plos.org%2Fplosone%2Fs%2Ffile%3Fid=ba62%2FPLOSOne_formatting_sample_title_authors_affiliations.pdf/1/010f01938a1ca323-6d26e037-a460-402c-9906-d1cf32822c62-000000/E2KscCelZX8wEZImZXojKBAO-ZYcSJ0C7w_XzajBVXs=187)

2. Please note that in order to use the direct billing option the corresponding author must be affiliated with the chosen institute. Please either amend your manuscript to change the affiliation or corresponding author, or email us at [plosone@plos.org](mailto:plosone@plos.org) with a request to remove this option.

Reply: We thank the Editor for the indication and included the necessary information in the Funding Information Section. The Institution that will fund the Open Access publication is the same where the Corresponding Author works, i.e., the University of Padova.

3. Please include your full ethics statement in the ‘Methods’ section of your manuscript file. In your statement, please include the full name of the IRB or ethics committee who approved or waived your study, as well as whether or not you obtained informed written or verbal consent. If consent was waived for your study, please include this information in your statement as well.

Reply: According to the Editor request, we included the information associated with the ethics committee and the study approvation number for the study in the Methods Section.

Changes made to the manuscript (Page 10): *“The study was conducted following the guidelines of the Helsinki Declaration and received approval from the local ethics committee (Etichs Committee for clinical sperimentation of Venice, approval n. 1013/IRCCSsancamillo). Exclusion criteria included history of epileptic seizures, neurological or psychiatric disorders, metal implants in their body, acoustic implants, non-removable piercings or metal accessories. Participants took part volountarly to the study and did not receive any form of compensation.”*

4. Please remove your figures from within your manuscript file, leaving only the individual TIFF/EPS image files, uploaded separately. These will be automatically included in the reviewers’ PDF**.**

Reply: We apologize for the mistake of including the images in the manuscript. According to the PLOS one guidelines, images are now included only as separate files. Each image is referred in the manuscript with the indication [Figure N.] to indicate the place the image should be placed.

**5.** We note that Figure 1 in your submission contain copyrighted images. All PLOS content is published under the Creative Commons Attribution License (CC BY 4.0), which means that the manuscript, images, and Supporting Information files will be freely available online, and any third party is permitted to access, download, copy, distribute, and use these materials in any way, even commercially, with proper attribution. For more information, see our copyright guidelines: <http://journals.plos.org/plosone/s/licenses-and-copyright>. We require you to either (1) present written permission from the copyright holder to publish these figures specifically under the CC BY 4.0 license, or (2) remove the figures from your submission.

Reply: The painting displayed in Figure 1 is distributed by the Metropolitan Museum of Art for unrestricted use under Creative Commons Zero (CC0), allowing to share, modify and publish this image with no restrictions. However, following the suggestion of Reviewer 2, we decided to remove the figure from the manuscript.

6. Please include captions for your Supporting Information files at the end of your manuscript, and update any in-text citations to match accordingly. Please see our Supporting Information guidelines for more information: [http://journals.plos.org/plosone/s/supporting-information](http://track.editorialmanager.com/CL0/http:%2F%2Fjournals.plos.org%2Fplosone%2Fs%2Fsupporting-information/1/010f01938a1ca323-6d26e037-a460-402c-9906-d1cf32822c62-000000/lvzIPZRdzbeeKwT3zBVpRvncL5OvajUGF857VhoWoSk=187).

Reply: According to the Editor’s suggestion, we included a caption for the Supporting Information file at the end of the manuscript.

Changes made to the manuscript (Page 42): *“****S1 Table. Correspondence between Destrieux regions and Yeo Networks.*** *Table displaying correspondences between each Region of Interest (Destrieux Atlas) and the corresponding Yeo Netwok. Coordinates indicate the centroid of each region.*

***S1 Fig. Comparison between InterNC and IntraNC for the seven Yeo networks in each condition.***

*Networks presenting significant differences between InterNC and IntraNC in the alpha band. Differences significant for p < .05 are displayed as black asterisks, whereas those significant for q < .05 are marked with a white diamond. The scale on the left represents t-values derived from statistical comparisons. DMN: Default Mode Network; DAN: Dorsal Attention Network; FPCN: FrontoParietal Control Network; LN: Limbic Network; SMN: SomatoMotor Network; VAN: Ventral Attention Network; VN: Visual Network.*

***S2 Fig. Comparison of InterNC and IntraNC for the seven Yeo network across the three conditions.*** *Comparison of Functional Connectivity in the three conditions in the alpha band. A. Comparison between the Monitoring-load and the Baseline conditions; B. Comparison between the Maintenance-load and the Baseline conditions; C. Comparison between the two PM conditions. Differences significant for p < .05 are displayed as black asterisks, whereas those significant for q < .05 are marked with a white diamond. The scale on the left represents t-values derived from statistical comparisons. DMN: Default Mode Network; DAN: Dorsal Attention Network; FPCN: FrontoParietal Control Network; LN: Limbic Network; SMN: SomatoMotor Network; VAN: Ventral Attention Network; VN: Visual Network.”*

Additional Editor Comments: Please provide details of the data acquisition parameters. For Freesurfer, please explain details of the preprocessing. For the whole manuscript, please be consistent about within- and between-network or inter/intra.

Reply: We thank the Editor for pointing out these issues. Regarding the preprocessing, we re-organized the information on MEG preprocessing, making them clearer and easier to follow, and included some missing information about MRI preprocessing. Regarding the terms “within” and “between”, they were replaced with the terms “intra” and “inter” throughout the whole manuscript to increase the consistency across the manuscript.

Changes made to the manuscript (Page 10-11): *“The MEG preparation session involved the placement of three head coils at anatomical landmarks (left and right preauricular points, nasion) for head localization and co-registration with anatomical images. Additionally, six amagnetic electrodes were used for auxiliary recordings: four were positioned around the eyes (two for detecting vertical eye movements and blinks, and two for horizontal eye movements), and two were placed on the left and right scapulas to capture cardiac activity. The positions of the head coils and the participant’s head shape were digitized using a Polhemus Fastrak system to enable precise co-registration. After the preparation, participants were seated comfortably inside a Faraday cage and given instructions about the experimental tasks. They were informed that they would be monitored and could communicate at any time during the session. Participants were also assured they could request a break or terminate the experiment if they felt uncomfortable in the MEG room. During data acquisition, MEG signals were recorded continuously at a sampling rate of 1200 Hz, with a hardware anti-aliasing low-pass filter applied at 300 Hz.*

*In the second session, neuroimaging data were collected using a 1.5 Tesla Philips Achieva MRI scanner. Specifically, a T1-weighted whole-head anatomical image was acquired for each participant (repetition time [TR]: 8.3 ms, echo time [TE]: 4.1 ms, flip angle: 8°, acquired matrix resolution [MR] = 288 × 288, slice thickness [ST] = 0.87 mm). These images were used to create individual head models for MEG source reconstruction. Preprocessing of MRI data was performed using FreeSurfer [44] and included skull stripping, segmentation of subcortical structures, cortical surface reconstruction, and the generation of smoothed pial and white matter surfaces.*

**Reviewer #1:** This study investigates prestimulus functional connectivity (FC) related to attention orientation in a prospective memory (PM) task using MEG. The authors differentiated the FC patterns between the monitoring and maintenance PM tasks during the prestimulus period. They observed increased inter-network FC of the dorsal attention network in the alpha band for the monitoring-load task, and increased FC of the ventral attention network with the frontoparietal and default mode networks for the maintenance-load task. These results demonstrate that different networks are engaged in PM depending on the specific features of the task. While I find this manuscript interesting, I have serious concerns about the analysis and the results. I hope the authors can address and clarify some of the ambiguities present in the current manuscript.

1. The authors introduce three models/theories: the preparatory attention and memory theory, the multiprocess framework, and the attention to the delayed intention model. These theories offer different interpretations of the mechanisms underlying PM. It would be helpful if the authors could clarify whether the FC data from the current study supports any of these theoretical accounts. Providing an integrated view that links the neural data with these theoretical frameworks could strengthen the manuscript, as the relationship between the cognitive theories and neural data is currently unclear.

Reply: We sincerely thank the Reviewer for this insightful comment. In the Discussion section of the manuscript, we addressed the interpretability of our functional connectivity (FC) findings within the context of the theoretical frameworks presented. Specifically, we discussed how our results could be interpreted as supporting the Attention to Delayed Intention (ATODI) model (see page 25) and the Multiprocess Framework (see page 26). To address the Reviewer’s concerns and improve clarity, we have expanded the Discussion section to include more explicit links between the FC results and these cognitive theories. We believe these additions not only clarify the connection between our findings and the theoretical frameworks but also strengthen the overall interpretability of our results. We trust that these revisions address the Reviewer’s concerns, but we are happy to make further modifications if needed.

Changes made to the manuscript (Page 25): *“The selective presence of this pattern in the Monitoring-load condition provides strong support for the Attention to Delayed Intention (ATODI) model [12]. Specifically, given that the instruction of this PM task enhanced the reliance upon strategic monitoring, the increased connectivity of the DAN regions with other networks may reflect enhanced top-down monitoring processes that prepare the system to actively search for the PM cue. This interpretation is further supported by the results of the comparison of alpha power across conditions, which revealed that in the Monitoring-load condition the DAN exhibited not only increased InterNC but also enhanced alpha activity—a marker of heightened suppression of irrelevant information and enhanced top-down processing [72] (Fig 7).”*

Changes made to the manuscript (Page 26): *“This finding may suggest that monitoring-enhancing PM instructions can induce a generalized "preparatory state" aimed at readiness to process forthcoming stimuli, in line with the Multiprocess Framework [7], which posits that PM task performance is supported by a dynamic interplay of automatic and controlled processes, depending on the nature of the task and environmental demands.”*

2. While I agree that MEG offers excellent temporal resolution compared to fMRI, I feel that this advantage could be better shown in the study. Could the authors provide more detailed temporal analyses of FC during the prestimulus interval? This could highlight the unique temporal dynamics captured by MEG.

Reply: We appreciate the Reviewer’s suggestion to conduct more time-locked analyses within the prestimulus interval to better showcase MEG's temporal precision. While we agree that additional approaches could have further highlighted the temporal dynamics of MEG, several theoretical considerations guided our decision not to pursue this in the current study. First, the inter-trial interval (ITI) was jittered to maintain participant vigilance and reduce similarities in processes across prestimulus intervals. This variability in timing complicates the use of precise time-locked analyses. Second, as discussed in the manuscript, defining a baseline interval to subtract average neural activity is a standard step for many analyses. While baseline normalization is not strictly mandatory, its absence is often discouraged in the literature. Without a clear stimulus to anchor epochs or define a baseline, time-locked analyses would have introduced interpretive challenges.

Instead, we focused on functional connectivity (FC) analyses, which are well-suited for investigating prestimulus activity without requiring time-locking to a specific event or baseline correction. Additionally, the unique temporal resolution of MEG allowed us to disentangle neural signals across distinct frequency bands (alpha, theta) and to analyze the patterns of connectivity within these bands, thereby leveraging MEG's strengths in capturing frequency-specific dynamics. We believe this approach aligns with the study's goals while adhering to methodological best practices.

3. The task description is somewhat unclear. To help readers better understand the experimental design and procedure, it would be beneficial if the authors provided visual illustrations, including timing and trial examples, in a figure.

Reply: We thank the Reviewer for this valuable suggestion. We agree that the original description of the experimental paradigm lacked clarity. In response, we have expanded the Task Procedure section to provide a more detailed account of the experimental design. Additionally, we have included a visual illustration of the paradigm to further enhance clarity and facilitate reader understanding. This illustration has been uploaded as Figure 1, and its corresponding caption has been incorporated into the manuscript.

Changes made to the manuscript (Page 13): *“****Fig 1. General structure of the experimental paradigm.*** *Independently from the condition, the strings of letters for the LDT were presented until a response was given, or for a maximum of 3000 ms. Hence, a blank screen was presented for 1000 ms, followed by a fixation cross displayed for an interval of 1500 ± 250 ms. A) In the Baseline condition, participants only had to perform the LDT. B) The structure of the Monitoring-Load condition was identical to the one of the Baseline, except for the addition of the instruction to press a different button when the syllable “PRA” was detected. C) In the Memory-Load condition, participants were instructed to press three different buttons at the presentation of three underlined words (AZZURRO, GRIGIO, and ROSSO).”*

4. Behavioural results are also crucial for understanding how the prestimulus neural data relates to task performance. Could the authors include these results in the manuscript?

Reply: We appreciate the Reviewer’s thoughtful comment regarding the importance of behavioral results for understanding how prestimulus connectivity relates to performance. The behavioral results for this dataset were analyzed and discussed in a previous publication, which we have referenced throughout the manuscript. To avoid redundancy, we did not analyze or discuss those results in this study. However, following a suggestion from Reviewer 2, we analyzed the relationships between behavioral data and prestimulus connectivity values. This new analysis revealed intriguing differences across conditions, offering further insights into the interplay between functional connectivity (FC) patterns and task performance. Specifically, we found that reaction times (RTs) were significantly correlated with FC values in the prestimulus alpha band during the two prospective memory (PM) tasks (Monitoring-Load and Maintenance-Load) but showed no significant correlation in the theta frequency band, nor in the Baseline condition. We have incorporated this additional analysis and interpretation into the revised manuscript (in the Abstract, Methods, Results, and Discussion sections) which now provides a comprehensive explanation of these relationships.

Changes made to the manuscript (Page 2): *“Additionally, response times were found to correlate with prestimulus alpha connectivity of different networks in the two conditions.”*

Changes to the manuscript (Page 16): “***Correlation between Behavioral and FC measures*** *Behavioral data were analyzed in a previous study, to which we refer the reader for further details [24]. In this study, we focused on analyzing the relationship between functional connectivity (FC) patterns and task performance to investigate whether distinct connectivity configurations in the prestimulus time window of different prospective memory (PM) tasks are associated with their behavioral outcomes. To achieve this, we paired the InterNC and IntraNC FC values of each network of interest with the corresponding Accuracy (ACC) and Response Times (RT) separately for each condition (Baseline, Monitoring-Load, and Maintenance-Load PM tasks) and frequency band (alpha, theta). Given that the Shapiro-Wilk test for multivariate normality indicated significant deviations from normality across all conditions (p < .001), we employed Spearman’s Rho, a non-parametric correlation measure, to assess the relationships between prestimulus FC and behavioral performance.”*

Changes to the manuscript (Page 21): *“****Correlation between Behavioral and FC measures***

*In general, all FC values—both InterNC and IntraNC—showed strong correlations with each other across all three conditions, both in the theta and alpha bands. Regarding their relationship with behavioral measures, significant correlations were found selectively in the alpha band, between FC measures and RTs (but not with accuracy). Notably, these correlations were found selectively in the two PM tasks, but not in the Baseline condition. In detail, InterNC and IntraNC values of the DAN and the VAN showed analogous associations with RTs in both PM conditions. However, the Monitoring-Load condition was characterized by a relationship between RT and InterNC of the FPCN (Rho = 0.466, p = 0.035), whereas in the Maintenance-Load condition RT resulted to correlate with IntraNC values of the DMN (Rho = 0.574, p = 0.007). Spearman’s Rho and p-values for each significant correlation are displayed in Table 1, separately for network and condition..”*

Changes made to the manuscript (Page 25): *“Subsequently, a correlation analysis was conducted to investigate the relationships between performance and these connectivity measures.” “A similar difference between frequency bands was also observed in the correlational analysis.”*

Changes to the manuscript (Page 27): *“Finally, the correlation analysis revealed significant associations between Response Times (RTs) and prestimulus alpha connectivity values. Specifically, both InterNC and IntraNC of certain networks positively correlated with RTs, consistent with prior studies reporting increased prestimulus alpha power in trials with longer response times [73,76]. Notably, these correlations were observed exclusively in the two prospective conditions (and not in the Baseline) suggesting that they may reflect task-specific preparatory mechanisms. In detail, the DAN and VAN consistently showed positive correlations with RTs across both PM tasks. In the Monitoring-Load condition, RTs also correlated with the InterNC of the FPCN, whereas the Maintenance-Load condition was characterized by a correlation between RTs and IntraNC within DMN regions. Considering the functional roles of the two networks—top-down control and communication among other networks for the FPCN and internal content processing for the DMN—the selective positive correlations of these two connectivity measures with RTs in the two prospective conditions (enhancing monitoring or maintenance demands, respectively) support the hypothesis of a dynamic interplay between networks in adapting to varying prospective task demands.”*

5. It is unclear which brain hemisphere's data were analysed for the FC analysis. Clarifying this would help readers understand the lateralization of the observed effects.

Reply: Due to the nature of prospective memory processes, which involve the activation of several bilateral regions (e.g., the anterior prefrontal cortices) as well as both hemisphere-specific regions, we included all 148 regions of the Destrieux Atlas in our analysis. This approach ensured that functional connectivity (FC) was assessed comprehensively across both the right and left hemispheres. We have updated the manuscript to explicitly state this choice in the Methods section to improve clarity for readers.

Changes made to the manuscript (Page 14): *“Namely, the brain surface was divided into 148 parcels (74 per hemisphere) according to the Destrieux Atlas”*

6. I have some questions regarding the FC analysis method. Could the authors clarify whether they extracted and normalized the correlation values before averaging and comparing the IntraNC and InterNC values among the conditions? It is important to ensure that any averaging of correlation values is done following proper normalization, as correlation is not a linear measure and cannot be simply averaged without introducing statistical inaccuracies.

Reply: We thank the Reviewer for raising this important point. We confirm that, prior to averaging, we meticulously followed the procedure proposed by Samogin et al. (2021). Specifically, we applied the Fisher Z-transformation (atanh) to the correlation values to ensure proper normalization. This transformation addresses the non-linear nature of correlation coefficients, making them suitable for averaging. Subsequently, we calculated the average connectivity values within (IntraNC) and between (InterNC) networks based on these normalized values. This approach minimizes potential statistical inaccuracies and allows for valid comparisons across conditions.

Changes made to the manuscript (Page 15): *“To ensure proper normalization, we applied the fisher z-transformation (atanh) to the correlation values prior to averaging.”*

7. Following up on the previous comment, could the authors elaborate on the statistical analysis conducted based on raw or normalized power values in Figure 7 (or on p.22)? I am uncertain whether statistical tests can be appropriately conducted on raw power spectra. A more detailed explanation would enhance the clarity of the results.

Reply: We sincerely thank the Reviewer for the valuable comment. Indeed, the initial analysis was conducted using raw Alpha power values. However, we acknowledge that this approach may not have provided reliable statistical results. To address this issue, we subsequently normalized the power values by applying a log-transformation, which ensures more appropriate and robust statistical analysis. This normalization step enhances the validity of our findings and allows for more accurate comparisons. The revised analysis uses a repeated measures ANOVA to assess differences across conditions and networks, which provides a comprehensive framework for identifying significant effects and interactions. Accordingly, we have updated the Methods section, the results, and the visual representation of our findings to reflect these changes.

Changes made to the manuscript (Page 22): *“Specifically, we calculated the raw power level for each node individually, applied a log-transformation to normalize the data, and then averaged it across the nodes within each network.”*

Changes made to the manuscript (Page 22): “*The repeated measures ANOVA (rmANOVA) on log-transformed alpha power revealed a significant main effect of Condition [F(2, 40) =5.000; p =.012; η_p_^2^=0.200] and a medium-sized interaction effect between Condition and Networks [F(6, 120) =2.252; p =.043; η_p_^2^=0.101] within the alpha band. Post-hoc analyses indicated that the primary differences were between the Baseline and Monitoring-Load conditions (p_bonf_ = .013), while the difference between the Baseline and Maintenance-Load conditions did not reach statistical significance (p_bonf_ = .077). Comparing the results separately within each network, the only difference in log-transformed alpha power resulted to be the difference between the Baseline and the Monitoring-Load condition within the DAN (p_bonf_ =.015). This result indicates that the DAN regions did not only display peculiar patterns of connectivity in the Monitoring-Load condition, but also enhanced levels of alpha power, a well-known marker of increased internal processing. The average alpha power of each network in the three conditions is displayed in Fig 7. Concerning the Theta band, no effects of Condition nor of its interaction with Network were found.”*

8. Could the authors specify the scale used in Figures 3 through 6? This would aid in the interpretation of the data presented.

Reply: The scales in Figures 3 through 6 represent the t-values obtained from the statistical analyses, with darker colors corresponding to greater statistical significance. Per the Reviewer’s suggestion, this information has been added to the captions of each figure. While we considered including this detail directly within the images, we decided to include it in the captions instead, as adding text to Figures 5 and 6 would result in writing that is too small to be legible. However, if the Reviewer prefers, we would be happy to add this information directly to Figures 3 and 4.

**Reviewer #2:** This study investigates the neural bases of prospective memory (PM) from a network connectivity perspective, using MEG data to assess functional connectivity (FC) across different brain networks under varying PM task demands. Specifically, the authors analyze two PM conditions—monitoring-load and maintenance-load—that emphasize either attentional monitoring or memory maintenance. By focusing on the prestimulus phase, the study identifies distinct connectivity patterns: increased inter-network FC of the Dorsal Attention Network (DAN) in the alpha band in the monitoring-load condition, and heightened connectivity of the Ventral Attention Network (VAN) with the FrontoParietal Control and Default Mode Networks (FPCN and DMN) in the maintenance-load condition. These findings suggest that top-down and bottom-up mechanisms are differentially engaged depending on PM task requirements.

While the study addresses an important and innovative approach to understanding PM processes through functional connectivity, several points require clarification and further elaboration to strengthen the manuscript.

Introduction

Page 3: Although original, the anecdote about Amedeo Modigliani’s paintings does not seem entirely appropriate for introducing the concept of prospective memory (PM) in a scientific context. I suggest reserving this example for a more popular science publication and omitting it from the scientific paper, as it may detract from the formality and focus of the study.

Page 4, line 82: In the retrieval phase, it should be noted that the associated intention must not only be correctly retrieved but also executed. Including an example to explain each phase would improve clarity.

Reply: We thank the Reviewer for the useful suggestions. We agree that the anecdote could detract formality from the study, even though it was originally included to put the focus on internal vs. external attention (the main manipulation in our study, within the PM domain). Therefore, we proceeded to remove it and changed the first part of the Introduction. In doing so, we also included explicit reference to the execution phase, which was missing from the description of the PM stages and included an example to improve the clarity of the differences between the four phases.

Changes made to the manuscript (Page 3-4): *“Prospective Memory (PM) refers to the ability to remember to perform an intention at the occurrence of a specific event or moment in the future [1]. PM processes are both frequent and essential in everyday activities [2]: common examples include remembering to take prescribed medication at the right time, updating a colleague after a meeting, or charging the phone upon finding a charger. Prospective remembering comprises several stages [3]: First, the intention must be formed and stored in memory along with the information associated with its retrieval (encoding phase). In the previous example, we might notice that our phone battery is low and decide to charge it as soon as possible. The stimulus or event associated with the intention (in this case, finding the charger) is defined as the PM cue. Subsequently, the intention must be retained in memory until the occurrence of the PM cue (maintenance phase). During this phase, individuals often engage in unrelated activities, known as ongoing activities (e.g., finishing our work activities) [4]. Simultaneously, attentional resources must be directed both to the environment (external attention), monitoring for the occurrence of the PM cue, and towards internal contents (internal attention) to uphold the intention in memory (e.g., “I need to charge the phone—where can the charger be?”) [5]. The balance between internal and external attention is essential for successfully retrieve the intention once the PM cue is detected (retrieval phase), comparing external stimuli with the predetermined idea of the PM cue. Finally, once the intention is correctly recalled from memory it can effectively be carried out (execution phase).”*

Page 5: When discussing The Multiprocess Framework, it’s important to address focal and non-focal cues and provide clear explanations, particularly since focality and salience are features manipulated in this experiment.

Page 5: “However, opposite patterns of activation have been observed in the medial and lateral aPFC and between the ventral and dorsal portions of the FPN during specific PM phases or in relation to PM task features.” This needs a brief explanation of how task features impact activation in these regions. Include a review of previous studies that have examined the role of the number of intentions, salience, and focality in PM, focusing on studies that explore these aspects at a neural level.

Reply: We agree with the reviewer that this information will enhance the quality and the clarity of the manuscript. A description of the effect of these features on the preferential activation of different brain regions was included in a previous version of the draft but was removed due to concerns on the length of the introduction. We proceeded to reinclude this part in the manuscript.

Changes made to the manuscript (Page 5): *“Briefly, focality refers to the extent to which the processing demands of the ongoing task overlap with those of the PM tasks (e.g., asking to detect a specific number while doing an arithmetical task vs. asking to detect all numbers starting with the letter “T”), whereas salience refers to the perceptual difference between the ongoing stimuli and the PM cue (e.g., a picture of a yellow taxi placed in a snowy landscape vs. one set against the busy traffic of New York City). Focal and salient stimuli have been consistently found to induce the activation of bottom-up processes (spontaneous retrieval), mostly relying on the ventral FPN, whereas nonfocal and non-salient stimuli induced the activation of the dorsal portion of the FPN [17,18].”*

Page 8. I recommend including a section titled "In the Present Study" to improve the flow and clarity of the text. In the hypotheses, clearly state the expected behavioral outcomes as well.

Reply: We thank the Reviewer for the valuable suggestion. In response, we have now divided the Introduction section in two parts, including the suggested “The Present Study” section for improved flow and clarity.

Changes made to the manuscript (Page 8): *“****The Present Study***

*This study employs a novel methodological approach proposed by Samogin et al. [38] to investigate functional connectivity (FC). Specifically, two measures—average within-network connectivity (IntraNC) and average between-network connectivity (InterNC)—were extracted for each network of interest, compared between networks, and analyzed across experimental conditions.”*

Materials and Methods

Page 10. Data Acquisition: Please specify any inclusion or exclusion criteria for participant recruitment and indicate whether participants received any form of compensation. Additionally, clarify how the sample size was determined. Conducting a power analysis to estimate the expected effect size would further support the adequacy of the chosen sample size.

Page 10. For MRI data, provide additional imaging parameters such as voxel size, repetition time, and echo time.

Reply: According to the Reviewer request, we provided additional information on the inclusion and exclusion criteria of the study, on the sample size definition, and the imaging parameters of the MRI acquisition.

Changes made to the manuscript (Page 10): *“The sample size was determined in the previous study employing this dataset [24] using G*Power to calculate the power analysis and was consistent with the numerosities of other studies investigating FC with the same methodologies [38,39]. The study was conducted following the guidelines of the Helsinki Declaration and received approval from the local ethics committee (Etichs Committee for clinical sperimentation of Venice, approval n. 1013/IRCCSsancamillo). Exclusion criteria included history of epileptic seizures, neurological or psychiatric disorders, metal implants in their body, acoustic implants, non-removable piercings or metal accessories. Participants took part volountarly to the study and did not receive any form of compensation.”*

Changes made to the manuscript (Page 11): *“In the second session, neuroimaging data were collected using a 1.5 Tesla Philips Achieva MRI scanner. Specifically, a T1-weighted whole-head anatomical image was acquired for each participant (repetition time [TR]: 8.3 ms, echo time [TE]: 4.1 ms, flip angle: 8°, acquired matrix resolution [MR] = 288 × 288, slice thickness [ST] = 0.87 mm).”*

Page 10-11. Task procedure: It is essential to clarify certain aspects of the experimental setup to improve transparency and reproducibility. What were the specific timings and intervals for each trial and block, including the presentation duration of task stimuli, inter-trial intervals, number of PM and ongoing trials per block, and any breaks between blocks? What mode and device were used to present stimuli, and what software was employed for stimulus delivery and response recording? Additionally, how were responses collected, and was response latency recorded? In the Maintenance-load block participants were instructed to maintain three different intentions in memory—what specifically were these intentions? Finally, did participants receive any practice or familiarization trials to ensure understanding of the tasks before starting the experimental blocks?

Reply: We thank the Reviewer for this valuable suggestion. Detailed information on the experimental paradigm is available in the two prior studies that employed the same methodology, as now stated more clearly at the beginning of the Task Procedure section. Nonetheless, we have incorporated the requested details into this manuscript, either as part of the text or through the visual representation of the experimental paradigm (Fig 1), which was also recommended by Reviewer 1. To maintain focus on the description of the functional connectivity (FC) analysis, we opted to provide a concise summary rather than including all the details from previous studies. However, we are more than willing to incorporate any additional information the Reviewer considers essential for improving the clarity and transparency of the experimental setup.

Changes made to the manuscript (Page 11): *“The experimental paradigm was the same employed in two previous studies [23,24] to which the reader is referred for further details. Briefly, the experiment was administered through the Psychopy software (version 1.85.2) [45] and consisted of three different blocks, each composed of 120 trials (a visual representation of the three blocks is displayed in Fig 1).”*

Changes made to the manuscript (Page 12): *“In both PM tasks, a total of ten PM cues were presented among the 120 trials (~ 10% of the total stimuli). PM cues were placed in pseudo-random positions, the first occurring after at least ten ongoing trials, and consecutive PM cues separated by at least eight ongoing trials. At the beginning of the experiment, a practice block involving only the ongoing task (five words/nonwords trials) was administered.”*

Changes to the manuscript (Page 13): *“****Fig 1. General structure of the experimental paradigm.*** *Independently from the condition, the strings of letters for the LDT were presented until a response was given, or for a maximum of 3000 ms. Hence, a blank screen was presented for 1000 ms, followed by a fixation cross displayed for an interval of 1500 ± 250 ms. A) In the Baseline condition, participants only had to perform the LDT. B) In the Monitoring-Load condition, participants were instructed to perform the LDT and to press a different button when the syllable “PRA” appeared. C) In the Memory-Load condition, participants were asked to perform the LDT and to to press three different buttons at the presentation of three underlined words (AZZURRO, GRIGIO, and ROSSO).”*

Page 12.” …considering only the ongoing trials with a correct response.” Please provide the average number of ongoing trials included per participant and block.

Reply: As previously stated, behavioral data were analyzed in a previous study (Cona et al., 2020). For this reason, in the methodology, we did not focus on the behavioral analysis or results. Additionally, accuracy reached a ceiling effect (a common phenomenon in the literature of PM) with high percentages of correct responses across all three conditions (Baseline: 97 ± 1.6%; Monitoring-Load: 96 ± 1.8%; Maintenance-Load: 98 ± 1.4%) and participants. Following your suggestion, we have included this information in the MEG preprocessing section, but limited it to the average of participants due to the low variability between participants. If the Reviewer believes the data from each participant would further improve the manuscript, we will proceed to include a table displaying this information.

Changes made to the manuscript (Page 13): *“(Baseline: 97%; Monitoring-Load: 96%; Maintenance-Load: 98%).”*

Page 12. Mention if any power analysis was conducted in the different analyses to ensure sufficient sensitivity for the paired t-tests, particularly given the multiple comparisons and FDR correction.

Reply: In the original version of the analysis, a power analysis of power spectra was not conducted. Following the Reviewer valuable suggestion, we have re-analyzed the data using log-transformed alpha power values, normalizing them to improve statistical validity. Additionally, we replaced the multiple paired t-tests with a repeated measures Analysis of Variance (ANOVA) to account for log-transformed alpha and theta power while applying corrections for multiple comparisons. We appreciate the Reviewer's insightful suggestion, which has enhanced the reliability and consistency of our findings.

Page 13-14. Please 1) include specific hypotheses regarding the IntraNC and InterNC comparisons within the hypotheses section of the experiment, and 2) explain how these processes of segregation and integration relate to PM processes within each of the networks of interest. This addition will clarify the expected outcomes and their connection to PM mechanisms across different networks.

Reply: We sincerely thank the Reviewer for this valuable suggestion, which has helped us significantly improve the clarity and comprehensiveness of the hypotheses section. In response to your comments, we have now included more explicit hypotheses regarding the IntraNC and InterNC comparisons within and between each of the networks of interest (DMN, DAN, FPCN, and VAN). Additionally, we have briefly discussed how the observed increases in IntraNC and InterNC relate to the underlying prospective memory (PM) mechanisms within each experimental condition.

Changes made to the manuscript (Page 9): *“Our first hypothesis predicts differing levels of FC between the Baseline and prospective memory (PM) conditions. In the Baseline condition, prestimulus activity is expected to reflect only the preparation for the ongoing task. In contrast, in the PM conditions, this interval also corresponds to the monitoring/maintenance phase. Therefore, we hypothesize higher levels of both IntraNC and InterNC in the alpha band in the PM conditions, reflecting the increased attentional demands directed toward both the intentions and the environment for detecting the PM cue, consistent with the ATODI model. The second hypothesis concerns distinct connectivity patterns characterizing the two PM conditions. In the Monitoring-load condition we expect increased involvement of the DAN, consistent with its role in the monitoring/maintenance phase of PM tasks. Specifically, heightened InterNC between the DAN and other networks in the alpha band is anticipated, reflecting the integration of attentional and cognitive control processes required for strategic monitoring. Conversely, DAN involvement is expected to not differ between the Baseline and the Maintenance-load conditions, as its PM demands induce reliance on spontaneous retrieval, a mechanism occurring after stimulus presentation [7]. Instead, the Maintenance-Load condition is predicted to show increased connectivity in the theta band within and between the DMN and VAN, reflecting higher memory demands and reliance on bottom-up processes.”*

Results

Page 17. What about the behavioral results? Please include them to confirm that the typical effects of a PM task are observed. Additionally, conducting a correlation analysis between behavioral outcomes and neuroimaging data could help interpret how functional connectivity influences performance in a prospective memory task. This would provide a more comprehensive understanding of the relationship between connectivity and behavioral execution in PM contexts.

Reply: We thank the Reviewer for this suggestion. As previously mentioned, the behavioral results for this dataset were analyzed and discussed extensively in a previous publication, which used time-frequency analysis to investigate neurophysiological correlates of the task. To avoid redundancy, we did not include them in this study. However, inspired by your comment, we conducted an additional correlation analysis to examine the relationship between behavioral outcomes and functional connectivity (FC). This analysis revealed that prestimulus FC measures were significantly associated with reaction times (RTs) during the two prospective memory (PM) conditions but not during the Baseline condition. Specifically, RTs showed selective correlations with InterNC and IntraNC of different networks across PM tasks, providing novel insights into the interplay between connectivity patterns and behavioral performance in PM contexts. We have incorporated these new findings into the revised manuscript.

Changes made to the manuscript (Page 2): *“Additionally, response times were found to correlate with prestimulus alpha connectivity of different networks in the two conditions.”*

Changes to the manuscript (Page 16): “***Correlation between Behavioral and FC measures*** *Behavioral data were analyzed in a previous study, to which we refer the reader for further details [24]. In this study, we focused on analyzing the relationship between functional connectivity (FC) patterns and task performance to investigate whether distinct connectivity configurations in the prestimulus time window of different prospective memory (PM) tasks are associated with their behavioral outcomes. To achieve this, we paired the InterNC and IntraNC FC values of each network of interest with the corresponding Accuracy (ACC) and Response Times (RT) separately for each condition (Baseline, Monitoring-Load, and Maintenance-Load PM tasks) and frequency band (alpha, theta). Given that the Shapiro-Wilk test for multivariate normality indicated significant deviations from normality across all conditions (p < .001), we employed Spearman’s Rho, a non-parametric correlation measure, to assess the relationships between prestimulus FC and behavioral performance.”*

Changes to the manuscript (Page 21): *“****3.3 Correlation between Behavioral and FC measures***

*In general, all FC values—both InterNC and IntraNC—showed strong correlations with each other across all three conditions, both in the theta and alpha bands. Regarding their relationship with behavioral measures, significant correlations were found selectively in the alpha band, between FC measures and RTs (but not with accuracy). Notably, these correlations were found selectively in the two PM tasks, but not in the Baseline condition. In detail, InterNC and IntraNC values of the DAN and the VAN showed analogous associations with RTs in both PM conditions. However, the Monitoring-Load condition was characterized by a relationship between RT and InterNC of the FPCN (Rho = 0.466, p = 0.035), whereas in the Maintenance-Load condition RT resulted to correlate with IntraNC values of the DMN (Rho = 0.574, p = 0.007). Spearman’s Rho and p-values for each significant correlation are displayed in Table 1, separately for network and condition..”*

Changes made to the manuscript (Page 25): *“Subsequently, a correlation analysis was conducted to investigate the relationships between performance and these connectivity measures.” “A similar difference between frequency bands was also observed in the correlational analysis.”*

Changes to the manuscript (Page 27): *“Finally, the correlation analysis revealed significant associations between Response Times (RTs) and prestimulus alpha connectivity values. Specifically, both InterNC and IntraNC of certain networks positively correlated with RTs, consistent with prior studies reporting increased prestimulus alpha power in trials with longer response times [73,76]. Notably, these correlations were observed exclusively in the two prospective conditions (and not in the Baseline) suggesting that they may reflect task-specific preparatory mechanisms. In detail, the DAN and VAN consistently showed positive correlations with RTs across both PM tasks. In the Monitoring-Load condition, RTs also correlated with the InterNC of the FPCN, whereas the Maintenance-Load condition was characterized by a correlation between RTs and IntraNC within DMN regions. Considering the functional roles of the two networks—top-down control and communication among other networks for the FPCN and internal content processing for the DMN—the selective positive correlations of these two connectivity measures with RTs in the two prospective conditions (enhancing monitoring or maintenance demands, respectively) support the hypothesis of a dynamic interplay between networks in adapting to varying prospective task demands.”*

Page 17. Include a brief discussion on the functional significance of these findings within each subsection, even if further elaborated in the discussion. This will provide readers with immediate insight into the relevance of the observed changes in the context of PM tasks.

Reply: According to the Reviewer’s suggestion, we included brief discussions on the functional significance of the findings within each subsection of the Results section. We agree that providing immediate feedbacks on the insights associated with our findings enhanced the manuscript’s clarity and accessibility.

Changes made to the manuscript (Page 18): “*While the higher InterNC of the VAN across all conditions may reflect bottom-up preparation to elaborate the upcoming stimulus, independently from its nature, the presence of the same effect over DAN regions selectively in the Monitoring-Load condition suggests a more top-down, strategical organization to detect less distinguishable PM cues.”*

Changes made to the manuscript (Page 19-20): “*and with the hypothesis of a generalized “strategic preparatory state” for detecting less salient PM cues” “both of which are associated with the processing of internal representations (Fig 5.B). Hence, this connectivity pattern may indicate an increased focus on maintenance processes and preparation to select the correct intention.” “This pattern of increased connectivity in the Monitoring-Load condition likely reflects heightened attentional resources directed toward the intention and allocated to upholding and refreshing the representation of a challenging-to-detect PM cue during the prestimulus time window, consistent with the engagement in strategic monitoring processes to prepare for intention retrieval. In contrast, in the Maintenance-Load condition, intention retrieval processes allegedly occur after stimulus presentation to recall the correct intention among multiple associated cues.”*

Page 22. Specify in the hypotheses section whether increases in alpha or theta power were expected in certain networks across conditions, based on prior literature or theoretical models of PM.

Reply: Following the Reviewer suggestion, we have now explicitly stated the expected frequency bands for each hypothesis concerning functional connectivity (FC) changes across conditions.

Changes made to the manuscript (Page 9): *“Therefore, we hypothesize higher levels of both IntraNC and InterNC in the alpha band in the PM conditions, reflecting the increased attentional demands directed toward both the intentions and the environment for detecting the PM cue, consistent with the ATODI model. The second hypothesis concerns distinct connectivity patterns characterizing the two PM conditions.” “Specifically, heightened InterNC between the DAN and other networks in the alpha band is anticipated, reflecting the integration of attentional and cognitive control processes required for strategic monitoring.” “Instead, the Maintenance-Load condition is predicted to show increased connectivity in the theta band within and between the DMN and VAN, reflecting higher memory demands and reliance on bottom-up processes.”*

Discussion

Page 23. How can you ensure that the prestimulus phase is not influenced by performance in the immediately preceding trial? Could there be differences in this phase resulting from a correct or incorrect response in the preceding ongoing trial? How might this be controlled?

Reply: We thank the Reviewer for this insightful comment. Different measures were taken to minimize and reduce the potential influence of performance in the preceding trial on the following. First, there was a minimum interval of 2250 ms between the response to the previous trial and the appearance of the next stimulus, as displayed in Figure 1. Namely, a blank screen was presented for 1000 ms after a response was given, followed by a fixation cross displayed for at least 1250 ms. The length of the prestimulus interval itself was selected for the specific purpose of reducing any confounding influence from the previous trial, also inclusing the appearance of the fixation cross occurring at least 1250 ms before the stimulus.

Additionally, while we agree with the Reviewer that the incorrect response on the previous trial could influence the subsequent monitoring/maintenance phase, we would like to point out that the high accuracy rates across participants (with very few incorrect responses across all conditions) substantially mitigate this potential issue. Therefore, we consider the impact of a prior error to be negligible.

Page 23. The PM cues in the different conditions (maintenance vs. monitoring) differ in focality and salience. While salience refers to the ease or difficulty with which a PM cue is perceptually detected, focality pertains to the similarity in processing between the PM cue stimulus and the stimulus in ongoing trials. Although both characteristics relate to detection and monitoring processes in PM, could each affect functional connectivity of these processes differently? Additionally, how might the results change if only focality were manipulated, given its direct connection to different retrieval mechanisms within the Multiprocess Framework?

Reply: The decision to manipulate focality and salience together was motivated by the aim of the study to create two PM tasks that differed strongly in terms of PM cue detection difficulty. This design was intended to enhance attentional requests and promote strategic monitoring in the Monitoring-Load condition while enhancing attention directed toward internal contents in the Maintenance-Load condition, in which participants were also required to maintain multiple intentions simultaneously.

Considering that salience is a perceptual property of the stimuli, while focality relates to the prospective task itself, it is likely that these two features influenced functional connectivity differently. Namely, we would expect salience modulation to mostly affect functional connectivity patterns involving the Ventral Attention Network (VAN), which supports bottom-up attention and the detection of relevant stimuli “popping out” among others. In contrast, focality is more closely associated with changes within the Frontoparietal Control Network (FPCN) and the Dorsal Attention Network (DAN), reflecting their roles in top-down cognitive control and strategic monitoring.

In a PM paradigm focusing only on focality manipulation, we would expect connectivity patterns to vary accordingly. A focal PM task would likely enhance connectivity within the FPCN and between the FPCN and VAN, reflecting the overlap between ongoing task processing and PM cue detection. Conversely, a non-focal PM task might strengthen connectivity between the FPCN and DAN, as strategic monitoring becomes more important to detect PM cues with less conceptually closer PM and ongoing tasks. Additionally, non-focal PM tasks can show reduced involvement of the VAN due to the increased difficulty in distinguishing the PM cue, leading to diminished reliance on bottom-up attentional mechanisms.

Page 26. "In contrast, in the Maintenance-load condition, the processes underlying intention retrieval occurred only after the detection of the PM cue, to retrieve the correct intention among multiple ones related to that cue"— I agree with this interpretation. To support this claim, is it possible to perform these post-stimulus analyses with the data you have?

Reply: We thank the Reviewer for agreeing with our interpretation. As explained in the manuscript, our study focuses on functional connectivity patterns in the pre-stimulus time window for several key reasons. First, our research question centers on the maintenance/monitoring phase of prospective memory. Second, behavioral responses were provided shortly after stimulus presentation (within 400 ms), which limited the power in the frequency bands of interest (Theta and Alpha), reducing their ability to show significant differences. Finally, a previous study had already analyzed alpha and theta power during the post-stimulus retrieval phase, using a different approach (Time-Frequency analysis). Given these constraints, it was not feasible to perform post-stimulus analyses with our data, as they did not provide the necessary temporal resolution or power to detect meaningful connectivity differences during retrieval.

Study Limitations and Future Perspectives

Page 28. There seems to be a potential contradiction here. In page 12, you mention segmenting the signal into epochs from 1.5 seconds before to 1.5 seconds after stimulus presentation, which would suggest that post-stimulus connectivity analysis is possible. However, in the limitations section, you note that post-stimulus analysis is not feasible due to participants' rapid responses (as early as 400 ms). Could you clarify this point? Specifically, is it possible to conduct any **preliminary post-stimulus analysis within the available 1.5-second epoch, even if limited?**

Reply: We apologize for the confusion and appreciate the Reviewer’s insightful comment. It is correct that, in our initial analysis, we segmented the signal into epochs from -1.5 seconds before to 1.5 seconds after stimulus presentation. These epochs were used to explore the time-frequency responses following stimulus onset (Cona et al., 2020). The larger epoch window was chosen to account for edge effects in both the interval of interest [0:1000 ms] and the baseline correction interval [-500:-200 ms].

In this second paper, however, we chose to focus exclusively on the prestimulus time window [-1000:0 ms] to investigate the PM processes related to the intention/maintenance phase independently from the stimulus-related responses, as we felt this was more relevant to our research question. While this approach was mentioned in the study's hypothesis, we acknowledge that it was not explicitly stated in the Methods section, and we sincerely apologize for this oversight.

Regarding the possibility of conducting Functional Connectivity (FC) analysis in the post-stimulus time window, we agree with the Reviewer that the rapid responses observed in some participants (as early as 400 ms post-stimulus) significantly reduced the time available for analyzing connectivity between stimulus onset and motor response. This 400 ms interval was deemed too short to perform reliable FC analysis in the frequency bands of interest (alpha and theta).

Additionally, we deliberately avoided conducting a second analysis on the same data used in our previous study, as the processes occurring after stimulus presentation may differ across experimental conditions (e.g., Monitoring-Load, Maintenance-Load, and Baseline). This further led us to believe that post-stimulus analysis would not be as relevant to the current investigation.

To summarize, while post-stimulus FC analysis is feasible with our data, we opted to focus on a different time window. We believe that post-stimulus analysis would be less interpretable and more challenging to justify methodologically in this context.

Changes made to the manuscript (Page 14): *“Connectivity values were calculated in the [-1000:0] time window of each trial and then averaged*, *separately for the theta (4-7 Hz) and alpha (8-13 Hz) frequency bands.”*

**Reviewer #3:** In this paper, authors investigated the effects of external (monitoring condition) and internal (maintenance condition) attention to detect a Prospective Memory cue on functional connectivity using MEG data. The neurosphysiological data demonstrated that the monitoring-load condition with respect to the baseline condition was characterized by increased inter-network FC of the DAN and that the maintenance-load condition (with respect to the baseline condition) was characterized by increased inter-network connectivity between the VAN and the FPN and the DMN. Both the modulations are observed in the alpha band. In addition, the authors hypothesized specific modulations in the theta band that however failed to reach significance after the correction of multiple comparisons for probable methodological concerns (i.e. the length of the prestimulus time window). The aim of the work is clear and well written.
Comments:
I understand why the authors focus the analysis on the networks involved in PM processing (i.e. DAN, DMN and VAN) and on the FPCN. However I believe that showing functional connectivity also within and between the other Yeo networks (not employed in this study) could strengthen the specificity of the results. If the authors believe that this whole-brain analysis would worsen the clarity of the text, they could add findings in supplementary materials.

Reply: We thank the Reviewer for this valuable suggestion. Initially, we considered conducting our analyses on the whole brain, encompassing all seven Yeo networks. However, to maintain focus and reduce the complexity of the analysis, we opted to concentrate on the four networks most closely associated with prospective memory processing. This decision was driven by the desire to reduce the number of regions (148 vs. 81) and correlations (21,756 vs. 6,480) while preserving statistical power. In response to the Reviewer’s suggestion, we conducted additional analyses including all seven Yeo networks. While the majority of the core findings remained robust (e.g., the increased alpha IntraNC-InterNC ratio in the DAN selectively in the Monitoring-Load condition), some results did not retain significance after correction for multiple comparisons (e.g., the increased IntraNC and InterNC connectivity of the DMN in the Maintenance-Load condition compared to Baseline). For this reason, we decided to maintain the original focus on the four networks of interest. However, we have now included the whole-brain analysis, encompassing all seven networks, as Supporting Information for transparency and to allow interested readers to explore these findings in greater detail.

Changes made to the manuscript (Page 14): “To assess the robustness of the results, the same analysis was also performed on all the seven Yeo networks, and the results are reported in Figs S1 and S2 (Supporting Information).”

Changes made to the manuscript (Supporting Information):

***“Figure S1. Comparison between InterNC and IntraNC for the seven Yeo networks in each condition.*** *Networks presenting significant differences between InterNC and IntraNC in the alpha band. Differences significant for p < .05 are displayed as black asterisks, whereas those significant for q < .05 are marked with a white diamond. The scale on the left represents t-values derived from statistical comparisons. DMN: Default Mode Network; DAN: Dorsal Attention Network; FPCN: FrontoParietal Control Network; LN: Limbic Network; SMN: SomatoMotor Network; VAN: Ventral Attention Network; VN: Visual Network.”*

**“*Figure S2. Comparison of InterNC and IntraNC for the seven Yeo network across the three conditions.****Comparison of Functional Connectivity in the three conditions in the alpha band. A. Comparison between the Monitoring-load and the Baseline conditions; B. Comparison between the Maintenance-load and the Baseline conditions; C. Comparison between the two PM conditions. Differences significant for p < .05 are displayed as black asterisks, whereas those significant for q < .05 are marked with a white diamond. The scale on the left represents t-values derived from statistical comparisons. DMN: Default Mode Network; DAN: Dorsal Attention Network; FPCN: FrontoParietal Control Network; LN: Limbic Network; SMN: SomatoMotor Network; VAN: Ventral Attention Network; VN: Visual Network.”*

In addition, I think that it would be very interesting to evaluate the directionality of these interactions in the frequency domain, for example using the directed transfer function that has been used on MEG data at the source level (e.g. using the Atlantis Processing Toolbox: atlantis.psychologia.uj.edu.pl, Spadone et al., 2021 Brain Connectivity). The authors could investigate this in future research and discuss the importance of examining directionality in communication.

Reply: We sincerely appreciate the Reviewer’s suggestion. As mentioned throughout the manuscript, the time-frequency data were previously investigated using the same dataset (Cona et al., 2020). However, we fully acknowledge the value of exploring the directionality of interactions in the frequency domain, as this could provide a deeper understanding of the neural dynamics underlying the observed connectivity patterns. The use of directed transfer function (DTF), particularly at the source level as suggested by the Reviewer and with tools such as the Atlantis Processing Toolbox (Spadone et al., 2021, Brain Connectivity), presents an exciting avenue for future research. We have added this approach to the Future Perspectives section of the manuscript, recognizing its potential to illuminate the directional flow of communication between neural regions, which is crucial for understanding complex cognitive processes like those involved in prospective memory. We are grateful for this suggestion and will consider it in our upcoming studies to further expand the scope of our investigation.

Changes made to the manuscript (Page 29-30): *“Additionally, future research should also explore the directionality of these interactions in the frequency domain, which may provide further insight into the mechanisms underlying PM tasks, employing approaches such as the directed transfer function, which has been effectively applied to MEG data at the source level [77]. Investigating the directional flow of information between neural regions might deepen our understanding of communication dynamics during PM processing.”*
